# Combination approaches to reduce weight-gain induced by antipsychotics

**DOI:** 10.1192/j.eurpsy.2021.111

**Published:** 2021-08-13

**Authors:** A. Hofer

**Affiliations:** Department Of Psychiatry, Psychotherapy And Psychosomatics, Medical University Innsbruck, Innsbruck, Austria

**Keywords:** Antipsychotic drugs, Treatment, Weight gain

## Abstract

Research demonstrates that the prevalence of overweight and obesity in the general population is increasing rapidly worldwide and that the environmental changes that have provoked these increases have also affected people with severe mental illness (SMI). Of note, obesity is two to three times more common among people with SMI and it contributes to a significantly reduced quality of life and to an increased morbidity and mortality rate in this population. The most important factor related to weight gain in people with SMI is the use of antipsychotic medication. Weight gain often occurs within 6-8 weeks after the initiation of antipsychotic treatment and may continue for at least 4 years. This can lead to non-adherence and risk of relapse. Next to behavioural interventions several pharmacological approaches have been investigated to deal with antipsychotic-induced weight gain. They target different receptor systems including dopaminergic, glutamatergic, serotonergic, adrenergic, opioid, and glucagon-like peptide 1 receptors. This symposium will provide an overview of the effectiveness of different add-on medications to treat weight gain in patients with SMI.

**Disclosure:**

No significant relationships.

